# From Weekly Journal to Monthly Review

**Published:** 1921-09-24

**Authors:** 


### The Hospital

The Workers' Newspaper of Administrative Medicine and Institutional Life. Administration. National Insurance and Health.

No. 1842, Vol. LXX. SATURDAY, SEPTEMBER 24, 1921. PRICE TWOPENCE

### FROM WEEKLY JOURNAL TO MONTHLY REVIEW.

With the present number The Hospital will cease to be published as a weekly newspaper in order that it may become a monthly journal under the title of The Hospital and Health Review. The new monthly journal will contain hospital news and criticisms as before, but it will enlarge its original sphere of interest by discussing, in addition to institutional matters, the multiform activities of public health. Preventive and curative medicine can no longer be divorced, and the science of public health is no longer one of drains and sanitation as it was when the original paper was founded. The hospitals are becoming more and more centres for the teaching and practice of health, no longer merely separate organisms but constituent parts of a co-ordinate body in which preventive medicine on the one hand, hospital teaching on the other, unite in a common effort for a common end. In consequence the activities of hospitals take on a new and wider perspective, which can best be presented in monthly review, wherein the whole movement can be brought into one focus.

A brief glance backward will best illustrate the motive for this change and the developments which have made it desirable. In 1885, when The Hospital was founded, the voluntary system was only beginning to emerge from a group of independent institutions whose common principle brought them into little harmony or inter-relation with each other. King Edward's Hospital Fund for London and the Royal National Pension Fund for Nurses had not been founded; the accounts of hospitals were not kept upon a uniform system; the League of Mercy was not established, and the Hospitals Association from which the British Hospitals Association sprang, was then in its infancy. At that time, too, the Royal Family was only beginning actively to identify itself with hospital work; and, despite the efforts of Florence Nightingale, nursing had not yet fairly won recognition as an organised profession for educated women. But the need for all these things was present to the mind of the Founder of this Journal, who had grown up with hospital work, and had never forgotten the needs of the provincial hospitals when he came to London. In the columns of this paper all these developments were advocated, and the story of their achievement was told. The paper also reflected the ideal of Sir Henry Burdett by advocating each of the changes mentioned, not only as the next step to be taken in the development of hospitals, but also from the belief that the voluntary system represented a theory of conduct for which the whole nation in and outside the hospitals was the better. Thus Hospital Sunday was supported not only as a source of revenue, but as the movement in which the motive of personal service to the sick was primarily emphasised. Hospital Sunday is the annual reminder that the support of voluntary hospitals is more than a business, and, if the voluntary system is to be maintained, its principle must be honoured for its own sake. If the motive power fails, the system which it created will collapse with it.

When the war came the response made by the hospitals showed that by 1914 the voluntary system had completed a chapter. The war brought the hospitals into the mid-stream of national life, and began to mould them, side by side with other movements, into a national system for the prevention and treatment of disease. To this extent the work, whose weekly history began in 1885, was accomplished. The period of individualism in hospitals and in medicine was over. The co-ordination of vigorous units into a national scheme of public health had begun. The country now has a Ministry of Health, and the nurses a College of Nursing and a State Register. The task for the future is to win the advantages of centralisation without sacrificing the voluntary impulse and the local independence which have governed us till now.

No longer are the voluntary hospitals a world to themselves. Side by side with them the public health medical service in its varied forms is in vigorous growth. Just as in the past the separate hospitals were helped to grow aware of their relation to each other by a journal which reflected the principle and aims of the voluntary system in general, so the need is now for a paper which may record and criticise that linking of hospitals and public health services which is the need of the present day. As things stand there is a lack of proper perspective. People cannot see the wood for the trees. The medical officer of health, the hospital superintendent, the general practitioner, the nurse, the health visitor are, at least mentally, more or less in the unco-ordinated relation in which the voluntary hospitals were a generation ago. Clearly, with their special interests and organs of opinion, they have not time nor energy to study one another's affairs and the mutual relation between them. In its new form, The Hospital and Health Review will make this possible, but if it is to do so a monthly issue is obviously the appropriate means. To neither hospitals nor the public services are diurnal matters so important as they were in the infancy of both. These are too numerous and too distracting, and may be safely left to the special journals which cater for each section every week. Hospitals and the public health services now need a critical and reasoned survey of their several but complementary activities; and thus it is that we believe our original readers will see in the new form of The Hospital a development of its traditions in the shape which the new importance of their work requires. As we said above, preventive and curative medicine cannot now be divorced. The Hospital and Health Review will provide a survey of both, and unite two streams which have run too long in parallel but unco-ordinated lines. The next issue will appear in the second half of October.

